# Socioeconomic status and race as social determinants of health to be considered in clinical use of pre-season vestibular and oculomotor tests for concussion

**Published:** 2020-10-07

**Authors:** Jessica Wallace, Phillip Worts, Ryan Moran, Justin Mason, Katherine K. Weise, Mark Swanson, Nicholas Murray

**Affiliations:** ^1^Department of Health Science, University of Alabama, 270 Kilgore Lane, Capital Hall 2106, Tuscaloosa, AL 35405, United States; ^2^Tallahassee Orthopedic Clinic 3334 Capital Medical Blvd Suite 600 Tallahassee, FL 32308, United States; ^3^Department of Nutrition, Food and Exercise Sciences, Florida State University, Tallahassee, FL 32308, United States; ^4^Florida State University Institute of Sports Sciences and Medicine Tallahassee, FL 32308, United States; ^5^Department of Occupational Therapy, University of Florida, 1225 Center Drive, Room 2119, Gainesville, FL 32611, United States; ^6^School of Optometry, University of Alabama at Birmingham, 1716 University Blvd, Henry Peters Building 506, Birmingham, AL 35233, United States; ^7^School of Community Health Sciences, University of Nevada, 1664 N. Virginia Street m/s 0274, Reno, NV 89557, United States

**Keywords:** race, socioeconomic status, concussion, vestibular/ocular motor screening, vestibular, King-Devick

## Abstract

**Background:**

Aside from racial and socioeconomic disparities in computerized neurocognitive testing and symptomology, there is a scarcity of research representing more diverse populations on other widely used tests for concussion, including vestibular and visual assessment.

**Aim:**

The aim of the study was to investigate if racial and socioeconomic differences exist on baseline vestibular/ocular motor screening (VOMS) and King-Devick (K-D) test performance in high school student-athletes.

**Methods:**

A total of 670 participants (66.1% White, 33.9% Black) with a mean age of 15.43±1.2 years were administered a baseline VOMS, average Near Point of Convergence (NPC) distance, and K-D test. The exposure variables included race (White or Black) and socioeconomic status (SES), defined as free and reduced lunch status (FRL or No-FRL). FRL status was determined by each participant’s school SES. The outcome variables consisted of baseline VOMS item symptom provocation scores, average NPC distance, and K-D baseline time. A series of Mann–Whitney *U* tests were performed for K-D baseline time, NPC distance, and VOMS items with FRL status or race as a between-subject factor. Two multivariable linear regressions were run to assess the association of (1) K-D baseline times using FRL, race, sex, and corrected vision as variables in the model and (2) average NPC distance using FRL, race, sex, and corrected vision as variables in the model.

**Results:**

When adjusting for multiple comparisons, FRL athletes had slower (worse) K-D times (*P*<0.001) than non-FRL athletes. Black athletes had significantly lower mean NPC distance compared to White athletes at baseline (*P*=0.02) and FRL status athletes reported a significantly greater (worse) mean symptom provocation following the visual motion sensitivity item on the VOMS (*P*=0.02); however, these findings were no longer significant following adjustments for multiple comparisons. No differences were noted for any remaining VOMS items. The first model explained 3.9% of the total variance of K-D baseline times, whereas the second model was not significant.

**Conclusions:**

Racial and SES differences existed on average NPC distance and the K-D test at baseline. Possible explanations for group differences may be neurobiological, anatomical, and/or disparity in nature. With a higher probability of undiagnosed and uncorrected vision impairment, vestibular dysfunction, and saccadic eye tracking deficits likely to be more apparent as a consequence of poverty or health inequities, it is important that healthcare providers, especially those that diagnose and treat concussions, understand that performance on the VOMS and K-D tests at baseline may be subject to sociodemographic factors of SES and race.

**Relevance for patients:**

To provide the most culturally competent care, clinicians should consider sociodemographic variables of race and SES as social determinants of health worthy of attention on objective and subjective measures of baseline concussion assessment.

## 1. Introduction

A concussion occurs following a direct or indirect traumatic force that is transmitted to the brain, which initiates pathophysiological processes (i.e., neurometabolic cascade) and results in transient brain dysfunction [1,2]. Patients may experience immediate or delayed onset of symptoms including headache, dizziness and fogginess, abnormal vestibular function, altered behavior, impaired cognition, sleep dysregulation, cardiac autonomic dysfunction, or visual dysfunction [3-5]. The diagnosis of a concussion is centered around a multifaceted assessment battery which includes symptoms, neurocognition, postural stability, and vision/vestibular assessments [1]. Concussion recovery may vary by age and prior history of concussion [6]; however, most adolescent concussions are believed to resolve in ≤4 weeks [3]. Unfortunately, a multitude of sociodemographic factors may affect clinical concussion diagnosis, treatment, and outcomes, which may be overlooked by clinicians.

Socioeconomic status (SES) (also known as socioeconomic position, sociodemographic characteristics, or sociocultural factors) has historically influenced clinical outcomes, dating as far back as the Pellagra epidemic in the 1900s [7,8]. Specific to concussion management, researchers are still reporting disparities in healthcare a century later, which disproportionally affects low-income patients and certain racial groups. Cognitive test performance [9], access to athletic trainers in secondary schools [10,11], access to specialized concussion care [12], and prolonged hospitalization following traumatic brain injuries are areas of inequity that warrant attention and must be addressed [13]. School SES, measured as a Title I classification [14], is a poverty indicator and the receiving of federal subsidies; however, these funds may not be prioritized for student-athlete health and safety. Further, 54% percent of high schools in the United States (US) meet school-wide Title I status and 52% of high school students in the US are eligible for free and reduced-price lunch (FRL) [15]. This proportion highlights the number of children nationwide who likely represent a lower SES. Many youths of low SES or a racial/ethnic minority are medically uninsured or insured by Medicaid [16]. This may limit their ability to seek specialized care for concussions. In fact, racial and ethnic minority youth sport participants are less likely to be diagnosed with a concussion in the emergency department compared to White youth [17]. Considered collectively, socioeconomically disadvantaged patients and those of non-White racial groups may not be positioned to attain optimal clinical outcomes if they suffer a concussion. In addition, if socioeconomically disadvantaged patients do seek medical attention following a concussion, it is uncertain if clinical diagnostics are capable of quantifying the influence of SES status on test results.

During various cognitive tests, processing speed [9,18-21], memory [9,18,20-22], and reaction time [18,20] have all shown to be susceptible to performance differences among racial groups at baseline or post-injury. In addition, total symptom reporting was significantly higher in Black athletes versus White athletes [23] and Black children reported higher cognitive-related symptoms post-concussion than their White counterparts [24]. Similarly, low SES groups performed worse on processing speed, memory, and reaction time tests and reported higher symptom scores [25]. Aside from racial and socioeconomic disparities in computerized neurocognitive testing and symptomology, there is a paucity of research representing more diverse populations on other widely used tests for concussion, including those that are designed to assess vestibular and visual dysfunction.

Concussion tests gaining rapid traction and clinical utilization, such as the King-Devick (K-D) test [26] or vestibular/ocular motor screening (VOMS) [27], have been used to assist clinicians in the detection of vestibular-ocular impairment following a suspected concussion, yet little is known about racial and SES differences on the test performance of the K-D and VOMS. However, on standard vision tests, there have been differences reported in prevalence rates of abnormal findings across racial groups [28-30]. A higher percentage of Black (14.1%) and Hispanic (14.2%) children failed one or more vision screening tests when compared to White children (11.0%) [28]. Likewise, the National Health and Nutrition Examination Survey estimates suggested that visual impairment was 2-3 times less prevalent in participants who reported a poverty income ratio that was more than 2 times above the poverty level versus their lower-income counterparts [31]. Further, increased odds of vestibular dysfunction were reported when comparing Black and Mexican American races/ethnicities to Whites [32]. Considering the VOMS and K-D both include vision performance metrics (e.g., Near Point of Convergence (NPC) in the VOMS and saccadic tracking in the K-D), the aforementioned clinical data would suggest that race and SES are worthy of considering when interpreting test data during a concussion evaluation. Therefore, the purpose of the current study was to investigate if racial and/or socioeconomic differences exist in performance on VOMS items, including average NPC distance and K-D test at baseline in high school student-athletes.

## 2. Methods

### 2.1. Design

This study utilized a cross-sectional design. The exposure variables were race (White or Black) and SES, defined as free and reduced lunch status (FRL or No-FRL). FRL status was determined by each participant’s school SES: Title I or non-Title I. Title I school SES was defined as high poverty, which includes schools where a greater percentage of students in attendance qualify for FRL, and non-Title I school SES was defined as low poverty included schools of which a greater percentage of students in attendance did not qualify for FRL [14]. Title I status was confirmed with administrators at each school district. The outcome variables consisted of baseline VOMS item symptom provocation scores, average NPC distance, and K-D baseline time.

### 2.2. Participants

A total of 868 high school student-athlete participants from 10 high schools in Northeast Ohio initially enrolled in the study. A total of 670 participants between the ages of 13 and 18 years (15.43±1.2 years) met the study’s inclusionary criteria. Any student-athlete diagnosed with a self-reported learning disability, concussion within the past 3 months, or any athlete who was missing baseline data on one of the two outcome measures (*n*=99) was excluded from the study. In addition, individual’s whose self-reported race was not White or Black (e.g., Hispanic/Latino, Asian, American Indian, and Pacific Islander) were excluded from the analysis (*n*=99) due to a low comparative sample size of additional minority races and ethnicities. A breakdown of participant demographics is provided in Table 1.

**Table 1:** Participant demographics.

	FRL status (%)		

	No FRL (*n*=232)	FRL (*n*=438)	Total (*n*=670)
Race			
White	202 (87.1)	241 (55.0)	443 (66.1)
Black	30 (12.9)	197 (45.0)	227 (33.9)
Sex			
Female	59 (25.4)	87 (19.9)	146 (21.8)
Male	173 (74.6)	351 (80.1)	524 (78.2)
Prescribed glasses or contacts			
Yes	113 (48.7)	187 (42.7)	300 (44.8)
No	119 (51.3)	250 (57.3)	369 (55.2)
Tested with corrective lens			
Yes	69 (29.7)	98 (22.4)	167 (25.0)
No – Not prescribed	119 (51.3)	251 (57.3)	370 (55.2)
No – Did not adhere to prescription	44 (19.0)	89 (20.3)	133 (19.9)
Age			
13	2 (0.9)	13 (3.0)	15 (2.2
14	48 (20.7)	129 (29.5)	177 (26.4)
15	54 (23.3)	95 (21.7)	149 (22.2)
16	70 (30.2)	109 (24.9)	179 (26.7)
17	52 (22.4)	83 (18.9)	135 (20.1)
18	6 (2.6)	9 (2.1)	15 (2.2)

Data reported as *n* (%). FRL: Free and reduced lunch status

### 2.3. Outcome measures

#### 2.3.1. VOMS tool

The VOMS was used to screen each student-athlete on eight individual items, including (1) smooth pursuits, (2) horizontal saccades, (3) vertical saccades, (4) convergence, (5) near point convergence distance (NPC), (6) horizontal vestibular-ocular reflex, (7) vertical vestibular-ocular reflex, and (8) visual motion sensitivity (VMS). Before the assessment, student-athletes reported a baseline rating for headache, dizziness, fogginess, and nausea on a scale of 0 (none)-10 (severe). After each item assessment, student-athletes rated each of the 4 symptoms again. Each VOMS item was administered to see if each subscale provoked any of the previously mentioned four symptoms. Symptom change scores were utilized for analyses and calculated by subtracting the pre-assessment symptom ratings from the post-assessment symptom ratings to reflect true symptom provocation of each item. NPC was collected using a tape measure and a Bernell fixation device. A total of three trials were collected by having each participant self-report diplopia or exophoria, measuring the distance with a tape measure, and then averaged. Normal NPC values are within 5 cm or less for children [33]. The averaged NPC values were utilized for analyses. High internal consistency has been previously reported for baseline NPC distance and K-D times in an adolescent student-athlete sample [34].

#### 2.3.2. K-D test

The K-D test is a rapid, number-naming tool requiring microsaccadic eye movements and reaction time. The K-D test measures the time it takes to read a series of numbers on a series of three test cards as quickly as possible, without making any errors. Scoring is calculated as the cumulative time that it takes to read three test cards, which get increasingly more difficult with each card, error-free. Each participant completed the K-D test twice, and the fastest of the two trials was then recorded as the participant’s baseline time. If a participant made an error on one of the 2 trials, he or she completed an additional trial(s) until an error-free attempt was completed. Each test was initiated with uniform directions as provided on the K-D test-card booklet followed by completion of the 1 demonstration card. High internal consistency (Cronbach a=0.92) has been recorded for baseline K-D times in youth populations [35]. Within this study, it is noted that the spiral-bound test card Version 1 of the K-D was used.

### 2.4. Procedure

Institutional Review Board approval was granted prior to any data collection. Further, all participants provided parental written consent before the start of the study. Participants were recruited from 10 high schools in the Northeast Ohio region by the principal investigator and athletic trainers employed at each high school. During pre-season baseline testing, student-athletes were individually administered the VOMS and K-D tests in a quiet classroom at their respective school. Each student-athlete was tested individually with a trained research team member. Data were collected from July 2016 through December 2018.

### 2.5. Statistical analysis

Before analysis, the dependent variables were screened for normality (i.e., skewness >2, kurtosis >9, Shapiro–Wilk *P*<0.05, and Q-Q plot observation). The Shapiro–Wilk test results indicated that the VOMS symptom provocation scores, NPC distance, and K-D test times (*P*<0.01) did not follow a normal distribution. Thus, a series of Mann–Whitney *U* test were run to assess group differences between race (Black or White) and FRL status (FRL, no-FRL) for pre-test and symptom provocation (i.e., change scores) on individual VOMS items along with NPC distance and K-D baseline times. Before modeling, outliers (>3 SDs) for K-D baseline time (*n*=9) and NPC distance (*n*=14) were removed. Two multivariable linear regressions were run to assess the association of (1) K-D baseline times using FRL, race, sex, and corrected vision as variables in the model and (2) average NPC distance using FRL, race, sex, and corrected vision as variables in the model. Due to autocorrelation (assessed through the Durbin Watson statistic), modeling was performed with general least squares and Nagelkerke’s pseudo *R*^2^ was reported [36]. Variable selection was informed by research noting that sex differences [37], and the use of corrective lenses, if prescribed, should be worn during the K-D test [38]. Finally, race may play a factor in the prescription and use of corrective lenses [39,40]; thus, it was important to examine the potential association between these factors for both analyses. An alpha level of 0.05 was set *a priori* and *P*-values were adjusted to control for multiple comparisons using the Benjamini–Hochberg procedure [41,42]. Variables with *P-*values significant after correction were indicated with an asterisk. All analyses were conducted in RStudio (RStudio, Boston, MA) with R version 4.0.2, using the nlme package.

## 3. Results

### 3.1. Vestibular/ocular motor and K-D baseline time differences

**Figure 1:**
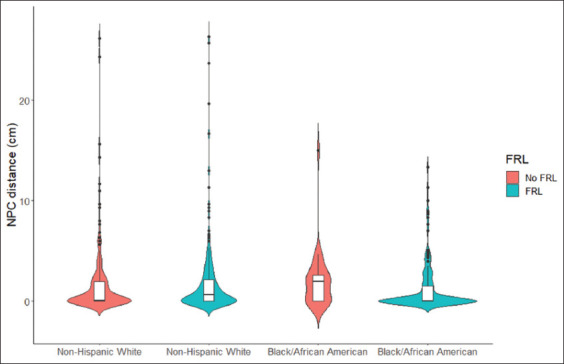
Violin plots of free reduced lunch (no or yes) by race and NPC distance (centimeters). Longer distances indicate worse performance.

A series of Mann–Whitney *U* test revealed small differences consistent with random sampling between race on pre-test symptoms of the VOMS (*U*=49571, *P*=0.41, Cohen’s *d*=0.01) or symptom provocation scores of any VOMS item (*U*=48867-50252, *P*=0.06-0.97); however, results were not statistically significant (Table 2). Regarding FRL status, no differences were observed between the FRL and non-FRL groups on pre-assessment symptoms of the VOMS (*U*=50642, *P*=0.11, Cohen’s *d*=0.14). Further, no statistically significant differences were observed on the VMS VOMS item (*U*=49152.0, *P*=0.02, Cohen’s *d*=0.11), with the FRL group reporting greater (worse) symptom provocation (0.10±0.9) than non-FRL (0.06±0.6). However, this VMS finding was no longer significant following the Benjamini–Hochberg procedure. There were no other statistically significant differences between symptom provocation change scores of any VOMS item between FRL status (*U*=49411-50664, *P*=0.11-0.77). Average NPC distance differed between race (*U*=55446, *P*=.02, Cohen’s *d=0*.16*)* but did not differ between the FRL and non-FRL groups (*U*=48584.0, *P*=0.31, Cohen’s *d*=0.04) (Figure 1). Refer to Table 2 for means and standard deviations. All medians and interquartile ranges were 0.00 for VOMS symptoms and NPC distance in the sample and between groups.

**Table 2:** Comparison of VOMS pre-assessment and symptom provocation change scores between race and FRL status*.*

VOMS item	White	Black	*P*	FRL	No FRL	*P*
Pre-test	0.19±1.3	0.19±0.9	0.42	0.25±1.5	0.07±0.5	0.11
Smooth pursuits	0.02±0.3	0.04±0.3	0.61	0.03±0.3	0.01±0.1	0.73
Horizontal saccades	0.04±0.4	0.07±0.5	0.14	0.06±0.5	0.03±0.2	0.60
Vertical saccades	0.08±0.6	0.11±0.6	0.44	0.10±0.7	0.07±0.5	0.77
Convergence	0.05±0.5	0.04±0.3	0.68	0.04±0.5	0.05±0.4	0.71
Horizontal VOR	0.08±0.6	0.08±0.5	0.97	0.08±0.6	0.08±0.5	0.70
Vertical VOR	0.08±0.6	0.05±0.4	0.96	0.05±0.5	0.09±0.5	0.31
VMS	0.09±0.6	0.20±1.1	0.06	0.10±0.9	0.06±0.6	0.03
Average NPC distance (cm)	1.82±3.7	1.27±2.3	0.02	1.59±3.2	1.72±3.3	0.32
K-D baseline time	49.60±7.9	49.24±8.7	0.55	50.19±8.3	48.19±7.7	<0.001*

Pre-test is non-provocation symptom tasks. *Significant after Benjamini–Hochberg procedure with false discovery rate 0.05. VOMS: Vestibular/ocular motor screening, FRL: Free and reduced lunch status, VOR: Vestibulo-ocular reflex, NPC: Near point of convergence, K-D: King-Devick, VMS: Visual motion sensitivity

**Figure 2:**
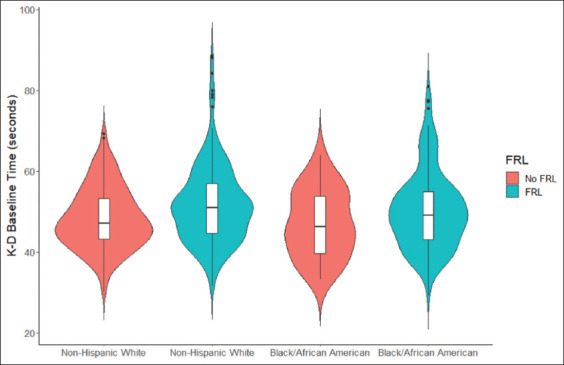
Violin plots of free reduced lunch (no or yes) by race and K-D baseline times (seconds). Longer times indicate worse performance.

The results of the Mann–Whitney *U* tests indicated that there was a significant difference between K-D baseline time and FRL (*U*=42513, *P*<0.01, Cohen’s *d*=0.32), where those who received FRL (50.19±8.33s) had slower (worse) overall K-D times compared to those not receiving FRL (48.19±7.70). Regarding potential racial differences, results of the Mann–Whitney *U* test indicated that there was not a significant difference between K-D baseline time and race (*U=*51685, *p*=0.55, Cohen’s *d*=0.04) (Figure 2).

### 3.2. Multivariable linear regressions

There was a dependence of residuals, as assessed by a Durbin–Watson statistic of 0.368. Thus, generalized least squares were used and results indicated that the model to investigate the association of K-D baseline times using FRL, sex, race, and corrected vision as predictors were significant (*F*(4,661)=8.94, Nagelkerke’s *R^2^*=0.039, *P*<0.001, adjusted *P*<0.001) with FRL (*P*<0.001), race (*P*=0.01), and sex (*P*<0.01) being the significant predictors. There was no evidence of multicollinearity, as assessed by VIF (VIF range 1.00-1.21). The overall model was able to explain 3.9% of the total variance of the dependent variable (Table 3).

**Table 3:** Multivariable linear regression results for K-D baseline time and average NPC distance measures (# Observations = 669 for both models).

	B	SE	95% CI	*P*
K-D baseline time				
Intercept	46.89	1.91	[43.15, 50.64]	
FRL	3.32	0.77	[1.80, 4.83]	<0.001*
Race	−1.99	0.81	[−3.58, −1.99]	0.01*
Sex	2.51	0.88	[0.79, 4.23]	<0.01*
Corrected vision	−0.31	0.44	[−1.17, 0.56]	0.49
Average NPC distance				
Intercept	2.31	0.72	[0.91, 3.72]	
FRL	0.09	0.29	[−.48, 0.66]	0.76
Race	−0.68	0.30	[−1.27, −0.68]	0.03*
Sex	0.30	0.33	[−0.34, 0.95]	0.35
Corrected vision	0.15	0.17	[−0.48, 0.17]	0.35

* Significant if the 95% CI does not include 0.00 and after Benjamini–Hochberg procedure with false discovery rate <0.05

There was a dependence of residuals, as assessed by a Durbin–Watson statistic of 0.008. Thus, generalized least squares were used and results indicated that the model to investigate the relationship of average NPC distance using FRL, race, sex, and corrected vision was not significant (*F*(4,656)=0.73, Nagelkerke’s *R^2^*=0.009, *P=*0.20, adjusted *P*=0.33), with race (95% CI=−3.58, −1.99, *P*=0.03) being the only significant predictor. There was no evidence of multicollinearity, as assessed by VIF (VIF range 1.00-1.21) (Table 3).

## 4. Discussion

The purpose of the current study was to investigate racial and socioeconomic differences in symptom provocation scores on VOMS items, average NPC distance, and K-D test total time at baseline in high school student-athletes. The hypothesis that there would be no differences between groups must be rejected. The primary findings of this study indicate that baseline administration of the VOMS and the K-D test exhibits racial and socioeconomic differences. First, the K-D baseline times were slower (worse) among those that receive FRL (50.19±8.3 s) when compared to those that do not receive FRL (48.19±7.7 s); however, no significant differences were noted by race. Further, a multivariable model to identify the association between K-D baseline time and independent variables suggests that FRL status and race are both independently associated with K-D baseline time when controlling for other independent variables. As a group, White individuals (1.82±3.7 cm) had greater (worse) average NPC distances when compared to Black individuals (1.27±2.3 cm) with a small effect size; however, when controlling for multiple comparisons, that difference was no longer significant. Greater symptom provocation reported on the VMS item was noted among those within the FRL group, but again that difference diminished after multiple comparison analyses. It should be noted that the average NPC distance was not outside the clinically accepted normal values for the potential diagnosis of concussion [27,43]. Possible explanations for group differences may be neurobiological, anatomical, and/or disparity in nature.

Upwards of 50% of the brain’s network is dedicated to vision [44] and adding a vision-based performance measure has enhanced the detection of concussion [45]. Two central findings from this study include a component of vision that appears to be deficient in high school student-athletes. First, a potential saccadic tracking skill deficiency was noted by slower K-D performance among those of FRL. Second, although within a clinically normal range, a potential racial difference in average convergence distance was noted. Both convergence insufficiency and deficient saccadic tracking skills are common binocular vision impairments and the rates of visual impairment are higher in 12-19 years old compared to other age groups, apart from those 60 or older [31].

Although a racial difference in average NPC distance was noted before multiple comparisons, the effect size was small. Black student-athletes had a lower NPC distance and, similarly, Black children have been shown to report fewer eye-related symptoms on the Convergence Insufficiency Symptom Survey (CISS) compared to White children [46]. These findings may allude to discrepancies or disparities in objective versus subjective measures for vision among racially diverse student-athletes that warrant further research. Within this study, a difference of approximately 0.6 cm is likely clinically insignificant and within normal limits of measurement error. However, these modest racial differences could be explained by the high potential for error of objective NPC distance using the nose as the measurement landmark specified with the VOMS. Further, NPC distance using the tip of the nose adds subjectivity to the measure and an influx of anthropometric errors can arise from anatomical variation in nose shape and length. Facial morphology studies using three-dimensional images have demonstrated racial differences in facial structure, including nose length between African Americans and the Welsh. African Americans displayed a less prominent nose and chin and more protrusive nasolabial fold when compared to the Welsh [47]. At present, there is a lack of racial or ethnic diversity in published VOMS norms, yet there is a substantial Black/African American presence in sport and higher-risk concussion sports. Suggestions to modify facial landmarks used for VOMS NPC measurements include using a less morphologically variable facial landmark, such as the lateral canthi (lateral confluence of upper and lower eyelid margins). Furthermore, assuring standardized administration methods may affect measurements [48]. Adding a more homogenous NPC measurement landmark to the VOMS like the lateral canthus may better align with measures used in eye care practices.

Previous research suggests that rates of uncorrected vision impairment are higher in low-income children due to poorer access to eye care services [39,40]. Further, hyperopia is suggested to be higher in non-Hispanic White children as compared to Black children [30], and uncorrected hyperopia has been negatively associated with progressive reading skills in youth [49] and adolescents [50] longitudinally. Binocular coordination of eye movements is essential for reading skills that also include the reading of numbers, as required by the K-D. Uncorrected vision problems can severely impact reading ability, and saccadic tracking skill deficits have been suggested to be a risk factor for poorer reading ability in adolescents [50,51]. Moreover, underdiagnosed and undertreated vision problems that influence the reading ability and academic performance often present as frequent eye rubbing or blinking, a short attention span, exo/esophoria, diplopia, losing one’s place when reading, tilting the head to one side when reading, or holding reading materials closer to the face [52]. As a vision-based tool that has gained traction in concussion detection, the K-D requires intact saccades and rapid-reading abilities; thus, the poorer performance among those of low SES can, in theory, be explained by higher potential for uncorrected vision impairments.

In addition to possible vision impairments leading to K-D reading deficits, the K-D was also suggested to be a cognitive-related tool due to pre-frontal cortex processing [53]. Rapid-reading ability can also be a consequence of reading skill level, which is linked to academic achievement differences between high SES and low SES groups. Reading skill ability and academic achievement gaps are widened by SES inequities and disparities determined by differences in the kind of school and classroom environment students have access to, resources within the home and neighborhood, and exposure to social capital necessary for success in school [54]. FRL is a widely-used proxy for SES, but SES is a stronger predictor of academic achievement for White students than students of a racial minority [54]. Moreover, the overall slower time to complete the K-D test could be also be due to potential reduced executive functioning and cognitive maturity of those with lower SES [55], as well as a greater likelihood of learning disabilities going undetected and undiagnosed among disadvantaged youth [56]. Results of our study parallel those by Weise *et al*. that found school-type differences on K-D performance among adolescents attending private versus public schools; however, school-type differences in the aforementioned study did not include a measure of SES such as FRL or Title I status [57]. In addition, the literature is sparse regarding how race may influence cognitive maturity or saccadic eye movements; however, the more complex the language [58] and the development of attentional capacity at a young age could influence eye movements and cognition in low-resourced areas [59]. However, these observations are purely speculative, given that the K-D test is not a direct measure of saccadic eye movements or cognitive function. It should also be noted that the significant differences in K-D test baseline results between FRL groups in this study are not believed to be clinically meaningful based on not exceeding the minimal detectable change threshold of 6.10 s [60] or the smallest reliable change index threshold of 3.61 s [61].

The VOMS symptom provocation score is an entirely subjective measure which is calculated by subtracting the reported score following each item by the pre-test value. This would lead the authors to believe that symptom provocation (i.e.) change scores were therefore subjected to the same limitations of previous symptom reporting tools [62-66]. Two main differences are that many scales use a 0-6 Likert scale while the VOMS uses a 0 to 10 Likert scale and the VOMS is designed to look at the change in symptoms to try an account for the individuality of reporting. Specific to race, Black student-athletes reported higher symptoms when compared to White student-athletes on a concussion symptom survey [23]; however, Black children have reported lower symptoms on the CISS [46]. Divergent responses on symptom reporting on concussion-specific versus vision-specific surveys prevent an obvious explanation for the findings in the current student. Furthermore, previous studies on standard vision exams [28-30] and vestibular testing [32] reported differences in abnormal findings and increased prevalence rates across racial and SES groups, but those results do not align with the results from the current study. One exception was the VMS item, in which the FRL group had a statistically significant greater symptom provocation score (0.16±0.9 vs. 0.06±0.6), although the findings were no longer significant after accounting for multiple comparisons. These results did not appear to amount to any appreciable clinical significance as the differences at baseline were not only below the clinical threshold but within the SD range of the groups, and the effect size (Cohen’s *d*=0.11) was below the “small” threshold.

The debate regarding baseline testing for intra-individual changes versus normative comparison has long been discussed. While the most recent consensus statement did not suggest mandatory baseline testing, there are data to suggest that, for some clinical tools, intra-individual comparisons provide superior utility versus normative comparisons [67-69] and because various types of athletes [70-72], or athletes from varying backgrounds perform differently on testing [25,73,74]; however, this is not always the case [75-78]. Our model predicted <4% of the variance in K-D test performance. These results indicate that performance is not well-predicted by race, sex, FRL status, and vision correction and provides support for baseline testing due to the inter-individual variability in our cohort.

As discussed previously, Title I schools provide funding to support their educational system but financial support does not always reach the sports medicine/athletic training departments on campus. Hence, while schools may appreciate the added benefit of baseline testing, it may not always be feasible. Fortunately, tools like the VOMS are publicly available and do not require a fee for use. However, there are always costs associated with baseline administrations, and while some tests are free, they are time-intensive for staff members and students, and IT infrastructure necessary to securely and easily access medical records should be considered.

### 4.1. Limitations

First, there is no perfect method to determine individuals’ SES, especially in a high school population, and thus, our findings are based on whether the student-athlete attended a school that met requirements for Title I or non-Title I status. This proxy for SES is widely utilized in educational research, but unfortunately, it is possible that some individual participants may belong to a different socioeconomic class that typically would not coincide with the need for FRL or Title I classification (e.g., affluent student at FRL school). However, we believe the quantity of those individuals is likely minimal. Furthermore, our data only included White and Black participants. Individuals from other racial or ethnic backgrounds (e.g., Asian, Hispanic/Latino) should be considered in future studies to add further diversity to test psychometrics and concussion literature. Finally, there was potential for inter-rater errors in test implementation and measurement. This study implemented the VOMS and K-D tests, which can be more affordable and easily implemented concussion screening assessments. Future research should examine if results are consistent with advanced laboratory-based vestibular and oculomotor tools (i.e., eye tracking). Finally, longitudinal studies are needed to determine modifying factors at post-concussion intervals (e.g., 3 days, 1 week, and 1 month), including race/ethnicity and SES.

## 5. Conclusions

As the clinical diagnosis and treatment of concussion actively continues to incorporate multisensory measures that include vestibular and vision-based oculomotor tools, respect for sociodemographic diversity is warranted. With a higher probability of undiagnosed and uncorrected vision impairment, vestibular dysfunction, and saccadic eye tracking deficits likely to be more apparent as a consequence of poverty or health inequities, it is important that healthcare providers, especially those that diagnose and treat concussions, understand that the VOMS and K-D tests at baseline may be subject to sociodemographic factors of SES and race. Further, there is a greater likelihood of learning disabilities going unrecognized and undiagnosed among disadvantaged youth, which may adversely affect performance on assessments such as the K-D [56]. Clinically, these results further emphasize the need for individual baseline assessments and growing need for generating more research on tools such as the VOMS and K-D in racially and ethnically diverse groups to add diversity to concussion literature. Pre-injury or baseline testing is commonly practiced by athletic institutions; however, in the absence of baseline data, clinicians rely on normative data for comparisons. Unfortunately, the utilization of normative data instead of pre-injury comparisons may increase false-positive [79] or false-negative rates [69] as also shown in the use of cognitive tests. If a patient is of a minoritized racial group or of low SES, normative data may not be an accurate or equitable reflection of expected performance post-injury or at the time of medical clearance. This is critical given the wide array of SES and racially diverse backgrounds that engage in an adolescent sport activity. It is recommended that when making interpretations on the potential clinical diagnosis of concussion using these tools, SES and race should be considered if comparing to norm-referenced data.

### Funding

This study was supported by a University Research Council grant from Youngstown State University.
